# Cognitive Improvement After Aerobic and Resistance Exercise Is Not Associated With Peripheral Biomarkers

**DOI:** 10.3389/fnbeh.2022.853150

**Published:** 2022-03-15

**Authors:** Soichi Ando, Takaaki Komiyama, Yukiya Tanoue, Mizuki Sudo, Joseph T. Costello, Yoshinari Uehara, Yasuki Higaki

**Affiliations:** ^1^Graduate School of Informatics and Engineering, The University of Electro-Communications, Chofu, Japan; ^2^Center for Education in Liberal Arts and Sciences, Osaka University, Osaka, Japan; ^3^Graduate School of Sports and Health Science, Fukuoka University, Fukuoka, Japan; ^4^Physical Fitness Research Institute, Meiji Yasuda Life Foundation of Health and Welfare, Shinjuku City, Japan; ^5^Extreme Environments Laboratory, School of Sport, Health & Exercise Science, University of Portsmouth, Portsmouth, United Kingdom; ^6^Faculty of Sports and Health Science, Fukuoka University, Fukuoka, Japan

**Keywords:** cognition, brain, reaction time, executive function, catecholamines

## Abstract

The role of peripheral biomarkers following acute physical exercise on cognitive improvement has not been systematically evaluated. This study aimed to explore the role of peripheral circulating biomarkers in executive performance following acute aerobic and resistance exercise. Nineteen healthy males completed a central executive (Go/No-Go) task before and after 30-min of perceived intensity matched aerobic and resistance exercise. In the aerobic condition, the participants cycled an ergometer at 40% peak oxygen uptake. In the resistance condition, they performed resistance exercise using elastic bands. Before and after an acute bout of physical exercise, venous samples were collected for the assessment of following biomarkers: adrenaline, noradrenaline, glucose, lactate, cortisol, insulin-like growth hormone factor 1, and brain-derived neurotrophic factor. Reaction time decreased following both aerobic exercise and resistance exercise (*p* = 0.04). Repeated measures correlation analysis indicated that changes in reaction time were not associated with the peripheral biomarkers (all *p* > 0.05). Accuracy tended to decrease in the resistance exercise condition (*p* = 0.054). Accuracy was associated with changes in adrenaline [r_*rm*_(18) = −0.51, *p* = 0.023], noradrenaline [r_*rm*_(18) = −0.66, *p* = 0.002], lactate [r_*rm*_(18) = −0.47, *p* = 0.035], and brain-derived neurotrophic factor [r_*rm*_(17) = −0.47, *p* = 0.044] in the resistance condition. These findings suggest that these peripheral biomarkers do not directly contribute to reduction in reaction time following aerobic or resistance exercise. However, greater sympathoexcitation, reflected by greater increase in noradrenaline, may be associated with a tendency for a reduction in accuracy after acute resistance exercise.

## Introduction

Acute aerobic exercise at light/moderate intensity improves cognitive performance (Chang et al., 2012; McMorris, 2021). The effects of acute resistance exercise on cognitive performance have received increasing attention, and recent reviews have suggested that resistance exercise also has the potential to improve cognitive performance (Soga et al., 2018; Wilke et al., 2019). It is widely speculated that an increase in arousal is responsible for these improvements in cognitive performance (Chang et al., 2012; Ando et al., 2020; McMorris, 2021). However, the mechanism(s) responsible for cognitive improvement following acute aerobic and resistance exercise remain unclear. Specifically, the role of peripheral biomarkers following acute physical exercise on cognitive improvement has not been systematically evaluated.

Adrenaline and noradrenaline are important for the adaptive response to physiological stressors through the activation of the sympathoadrenomedullary system (Danese et al., 2018). In this context, there is some evidence in the literature that acute physical exercise increases circulating catecholamine concentrations (Pontifex et al., 2019; McMorris, 2021). Although peripheral adrenaline and noradrenaline do not easily traverse the blood-brain barrier (Cornford et al., 1982), increased circulating adrenaline and noradrenaline activate β-adrenoceptors on the afferent vagus nerve, which terminates in the nucleus tractus solitarii (NTS) within the blood-brain barrier (McGaugh et al., 1996). Noradrenergic cells in the NTS also project to the locus coeruleus and stimulate noradrenaline synthesis and release to other regions of the brain (McMorris, 2016). Thus, increased circulating adrenaline and noradrenaline may, at least in part, lead to an improvement in executive performance.

Blood glucose is the primary source of energy for the brain (Gold, 1995) and recent studies indicate that increased blood lactate after high-intensity interval exercise is associated with attentional or executive performance (Tsukamoto et al., 2016; Hashimoto et al., 2018; Herold et al., 2022). These findings suggest that enhanced lactate metabolism may contribute to executive improvements. However, the role of blood lactate following acute light intensity aerobic and resistance exercise is relatively unknown. Similarly, in response to acute stress, cortisol is released by the adrenal cortex and is regulated by the hypothalamic–pituitary–adrenal (HPA) (Costello et al., 2018). Tsai and colleagues reported that alterations in cortisol level after resistance exercise are associated with electrophysiological activity (i.e., P3 amplitude) (Tsai et al., 2014). These findings suggest that alterations in circulating cortisol level could be linked to executive performance following acute physical exercise.

Acute resistance exercise increases peripheral insulin-like growth hormone factor 1 (IGF-1) (Rubin et al., 2005; Rojas Vega et al., 2010; Tsai et al., 2014, 2018). Acute aerobic (Huang et al., 2014; Tsai et al., 2018), and resistance (Marston et al., 2017) exercise increases peripheral brain-derived neurotrophic factor (BDNF). Rodent studies suggest that increases in IGF-1 are related to improvements in learning and spatial memory after a period of training (Ding et al., 2006; Cassilhas et al., 2012). In contrast to adrenaline and noradrenaline, BDNF crosses the blood–brain barrier in both directions (Pan et al., 1998) and it has also been speculated that elevated BDNF might contribute to improvements in executive performance/memory after acute physical exercise (Ferris et al., 2007; Griffin et al., 2011; Piepmeier and Etnier, 2015; Borror, 2017). Indeed, improvement in executive performance was associated with exercise-related changes in BDNF (Hwang et al., 2016). However, IGF-1 and BDNF appears to play a crucial role in angiogenesis, synaptogenesis, and neurogenesis following long-term exercise (Cotman and Berchtold, 2002; Voss et al., 2011; Nieto-Estevez et al., 2016) and further studies are necessary to understand whether transient increases in IGF-1 and BDNF contribute to an improvement in executive performance following acute physical exercise.

Acute light aerobic exercise has been suggested to improve executive performance (Chang et al., 2012), and peripheral adrenaline and noradrenaline concentrations increase after acute light aerobic exercise (i.e., 40% maximal oxygen uptake) (McMurray et al., 1987). If changes in peripheral biomarkers, such as adrenaline and noradrenaline, are associated with alterations of the performance of executive functioning, it seems reasonable to hypothesize that an association between peripheral biomarkers and executive functioning can be observed even after light-intensity physical exercise. Lactate production in response to acute exercise depends on type of exercise (i.e., aerobic or resistance) (Hashimoto et al., 2021). Acute light aerobic exercise does not increase blood lactate concentration (Ivy et al., 1980). Conversely, resistance exercise at low intensity using elastic bands appear to increase blood lactate (Yasuda et al., 2014). Furthermore, an acute bout of resistance exercise, but not aerobic exercise, is a physiological stimulus for acute increases in IGF-1 (Gregory et al., 2013). These suggest that changes in some peripheral biomarkers (e.g., lactate and IGF-1) may be greater after resistance exercise relative to aerobic exercise. Hence, comparing the effects of aerobic and resistance exercise on executive performance and peripheral biomarkers can help to elucidate the common or divergent molecular mechanisms driving changes in cognitive performance after different types of acute physical exercises (i.e., aerobic vs. resistance exercises).

Accordingly, this study sought to investigate the relationship between peripheral circulating biomarkers and executive performance following acute, intensity matched, aerobic and resistance training exercise. We tested the hypothesis that: (i) acute aerobic and resistance exercise would improve executive performance, (ii) peripheral biomarkers would be associated with this performance, and (iii) the differential effects of aerobic and resistance exercise on peripheral biomarkers would delineate the specific contribution of these biomarkers to improvement in executive performance.

## Materials and Methods

### Participants

Nineteen healthy males volunteered to participate in this study [age: 22.5 ± 2.3 year; body weight: 1.71 ± 0.06 m; body mass: 66.2 ± 7.6 kg; peak oxygen uptake (V̇O_2*peak*_): 48.2 ± 7.1 ml kg min^–1^]. All participants were physically active and met the following criteria: (i) right handed as assessed by the Edinburgh Handedness Inventory (Oldfield, 1971); (ii) low-risk status for physical exercise-related adverse events assessed by Physical Activity Readiness Questionnaire (Warburton et al., 2011); and (iii) no history of cardiovascular, cerebrovascular, or respiratory disease (self-report). All participants gave their written informed consent prior to participation. Sample size was calculated using G-power (version 3.1) (Faul et al., 2009) based on our pilot data which suggested that a reduction in reaction time (RT) after aerobic exercise at 40% V̇O_2*peak*_ was ∼28 ms (Cohen’s day effect size of 0.4). Accordingly, a minimum of 15 participants were required to achieve a power of 80% with an alpha of 0.05. The participants were instructed to abstain from any strenuous exercise for 24 h and food, caffeine and alcohol for 12 h prior to the laboratory visit. All experimental procedures adhered to the standards set by the latest revision of the declaration of Helsinki, except for registration in a database, and were approved by the ethics committee of Fukuoka University (2015-09-01).

### Cognitive Task

Central executive function was assessed using a Go/No-Go task. The task was completed on a laptop computer (Let’s note CF-R4, Panasonic, Osaka, Japan) placed 80 cm from the participants. The participants performed the cognitive task sitting on a chair. The details of the cognitive task are described in detail elsewhere (Ando et al., 2013). Briefly, the participants were required to either respond (Go trial) or not (No-Go trial) according to the stimulus. A shift-key on the keyboard was used to perform the cognitive task. The participants pressed the key using the right index finger. A total of 30 trials were completed. Both RT and accuracy of the task were used to assess executive performance. Omitting a response in a Go trial or performing an incorrect response in a No-Go trial was regarded as an error trial. Accuracy of the task was calculated as number of correct response/total number of trials.

### Experimental Procedure

This study employed a within-participants pre-test post-test crossover comparison in line with the taxonomy provided by Pontifex et al. (2019). The experiment was performed over three non-consecutive days with intervals of at least 3 days between experimental sessions. Throughout the experiment, the ambient temperature was maintained at 22°C and the relative humidity was controlled approximately at 50%. On the first day, the participants practiced the cognitive task until they were familiar with the task to minimize the impact of a learning effect. Thereafter, the participants performed a maximal exercise test to exhaustion on a cycle ergometer (75XLII, COMBI Wellness, Tokyo, Japan). After a warm-up period at 10 W for 1 min, the test was initiated with 20 W increments every minute in a ramp manner. Participants were instructed to maintain a cadence of 60 revolutions per min (rpm), and the test was terminated when they were unable to maintain a cadence of >40 rpm. Minute ventilation, oxygen uptake, fraction of end-tidal CO_2_, and O_2_ were recorded using a gas analysis system (ARCO-2000, ARCO System, Chiba, Japan), and V̇O_2*peak*_ was determined as the highest value attained over the course of 1 min. Exercise intensity at 40% V̇O_2*peak*_ (90 ± 13 watts) was subsequently calculated for aerobic exercise.

On the second and third days of the experiment, the experiments were performed on the same time to minimize circadian effects. At the beginning of the experiment, venous blood sample was collected from the antecubital vein for the analyses of adrenaline, noradrenaline, cortisol, IGF-1, and BDNF analysis. The left earlobe was pricked with a safety lancet and 2 μL capillary blood was collected for glucose and lactate analysis. Systolic blood pressure and diastolic blood pressure were measured from the right arm in a sitting position (HEM-705IT, Omron Healthcare, Kyoto, Japan). Mean arterial pressure (MAP) was subsequently calculated. Then, the participants performed the first cognitive task. After the cognitive task, the participants performed either aerobic or resistance exercise for 30 min. The order of exercise type was randomly counterbalanced. Immediately after the exercise, venous and capillary blood samples were collected, and blood pressure was measured. Then, the participants performed the second cognitive task.

Several studies have compared the effects of aerobic and resistance exercise on cognitive performance using a randomized crossover design (Pontifex et al., 2009; Alves et al., 2012; Harveson et al., 2016; Dunsky et al., 2017). However, exercise intensity is one of the key factors that determine the exercise-cognition interaction (Chang et al., 2012) and these previous studies did not attempt to match heart rate (HR) between the aerobic and resistance exercise. This is most likely attributed to the challenges associated with matching HR during both aerobic and resistance exercise. Thus, following extensive pilot tests, we attempted to match ratings of perceived exertion (RPE) (6–20 Borg scale) (Borg, 1975) between aerobic and resistance exercise. In the aerobic exercise condition, the intensity was set at 40% V̇O_2*peak*_. Mean HR corresponded to 58.7 ± 6.0% age-predicted maximal HR in the aerobic condition and 52.7 ± 7.5% in the resistance condition. Thus, exercise intensity of the aerobic exercise was considered light according to the ACSM guidelines (Riebe et al., 2018). In the resistance condition, the participants performed resistance exercise using elastic bands (Spoband 55, YKC, Tokyo, Japan). We used elastic bands for the resistance exercise as they are low-cost, portable, have a low risk of injury, and are easily accessible. The findings may be practically useful for exercise prescription at home, or in other settings. The validity of intervention was confirmed by a previous study (Roh et al., 2020). The resistance exercise program was designed to use all major muscle groups (Hofmann et al., 2016). The resistance of the band was 10.3 kg when the length of the band was doubled. The participants performed an exercise program incorporating 42 difference exercises (see Supplementary Table 1). This program targeted the following muscle groups: shoulder, chest, back, arms, abdomen, hip, and legs, and included three types of muscle contractions (concentric, eccentric, and isometric) and both multi- and single joint movements. After brief instruction for each program, and participants completed 10 reps of each exercise. The duration of the exercise program was 30 min.

### Measurement

Heart rate was recorded continuously using a heart rate monitor (RS800CX; Polar Electro Oy, Kempele, Finland). RPE was recorded before and immediately after exercise. Blood glucose concentration was measured by glucose oxidase method using blood glucose monitor (Glutest Ace, Sanwa Kagaku, Nagoya, Japan). Blood lactate concentration was determined by the lactate oxidase method, using an automated analyzer (Lactate Pro, Arkray, Kyoto, Japan). Blood sample volume was ∼15 ml for each measurement, and both plasma and serum samples were collected. Plasma samples were obtained from heparinized blood samples by centrifugation at 3,000 rpm for 15 min and stored at −80°C until analysis. Plasma adrenaline and noradrenaline concentrations were determined using a high-performance liquid chromatography system (Shimadzu, Kyoto, Japan). Serum samples were obtained from the venous blood by centrifugation at 3,000 rpm for 15 min and stored at −80°C until analysis. Serum cortisol was measured by commercial radioimmunoassay kit (Immunotech, Marseille, France). Serum IGF-1 concentration was determined using an immunoradiometric assay (IGF-1 IRMA Daiichi, TFB, Tokyo, Japan) and a Wallac 1460 Gamma Counter (Wallac, Turku, Finland). Serum BDNF concentration was measured using the Quantikine Human BDNF Immunoassay (R&D systems, Minneapolis, United States). Due to collection issues resulting in too small sample volumes, the concentration changes of BDNF from two participants (one in the aerobic condition and one in the resistance condition) could not be determined. Adrenaline, noradrenaline, cortisol, IGF-1, and BDNF concentrations were measured at the SRL Clinical Laboratory (Tokyo, Japan). All samples were analyzed in duplicate. Intra- and inter-assay coefficients of variance were 2.8 and 2.6% for adrenaline, 1.0 and 1.4% for noradrenaline, 1.7 and 1.6% for cortisol, and 3.5 and 3.0% for IGF-1.

### Data and Statistical Analysis

The distribution of data was assessed using descriptive methods (skewness, outliers, and distribution plots) and inferential statistics (Shapiro-Wilk test). We performed a two-way repeated-measures ANOVA [Exercise Type (aerobic, resistance) × Time (pre, post)] for all variables, followed by Bonferroni-corrected paired *t*-tests for normally distributed data or the Wilcoxon signed rank test for non-normally distributed data. All the RT values were plausible, and within the expected ranges, based on our previous studies (Ando et al., 2013; Komiyama et al., 2015). Effect sizes are presented as partial eta-squared (η_*p*_^2^) in the main effects and interactions. Statistical analyses were performed using SPSS (Statistical Package for the Social Sciences) version 25.0 (SPSS Inc., Chicago, IL, United States). We also performed repeated measures correlation analysis (Bakdash and Marusich, 2017; Marusich and Bakdash, 2021) between executive performance (both RT and Accuracy) and peripheral biomarkers. Raincloud plots were created using the JASP version 0.16 (JASP team, Amsterdam, Netherlands). Data are expressed as mean ± SD or median (interquartile range). The significance level was set at *p* < 0.05.

## Results

**FIGURE 1:**
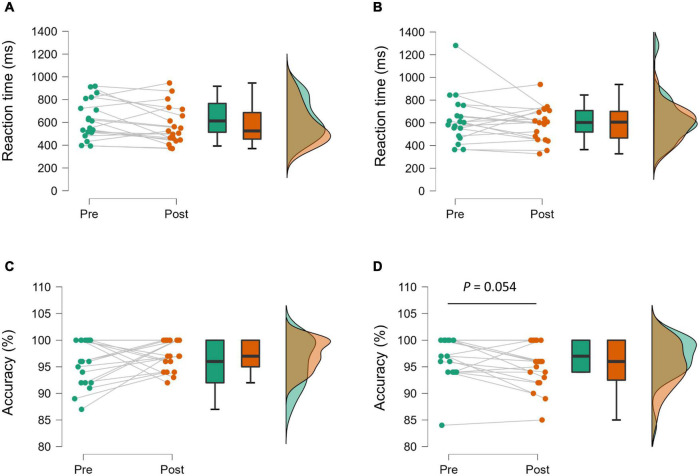
Raincloud plots showing the distribution of reaction time [**(A)** aerobic condition, **(B)** resistance condition] and accuracy of the Go/No-Go task [**(C)** aerobic condition, **(D)** resistance condition]. Plots and lines represent individual data (left). The lower and upper hinges on the boxplots represent the first and third quartile with the median (center). Illustration of data distribution (right).

Figure 1 illustrates RT and accuracy of the Go/No-Go task. A significant main effect of Time was observed in RT [*F*_(1_,_18)_ = 5.02, *p* = 0.038, η*_*p*_^2^* = 0.22]; however, no effect of Exercise Type [*F*_(1_,_18)_ = 0.07, *p* = 0.792, η_*p*_^2^ = 0.004], or interaction [*F*_(1_,_18)_ = 0.06, *p* = 0.812, η*_*p*_^2^* = 0.003] was observed. There were no significant main effects of Exercise Type [*F*_(1_,_18)_ = 0.41, *p* = 0.530, η_*p*_^2^ = 0.02] and Time [*F*_(1_,_18)_ = 0.01, *p* = 0.939, η_*p*_^2^ = 0.000] on the accuracy of the Go/No-Go task. However, there was a trend toward a significant interaction effect [*F*_(1_,_18)_ = 4.41, *p* = 0.050, η*_*p*_^2^* = 0.20].

Table 1 summarizes HR, RPE, MAP, and peripheral biomarkers. For HR, we found a significant interaction between Exercise Type and Time, and HR was significantly greater during aerobic exercise than that during resistance exercise (*p* = 0.004). RPE increased after aerobic and resistance exercise. Both adrenaline and noradrenaline increased after both aerobic and resistance exercise, but there were no differences between the modalities (all *p* > 0.05). Glucose significantly decreased after aerobic and resistance exercise (main effect of Time, *p* = 0.001). Although lactate increased after both aerobic (*p* = 0.045) and resistance (*p* < 0.001) exercise, it was higher after resistance exercise (*p* < 0.001). Cortisol decreased after aerobic and resistance exercise (main effect of Time, *p* = 0.002). IGF-1 was elevated following resistance exercise (*p* = 0.003), but not after the aerobic exercise (*p* = 0.36). There was a trend for BDNF to be higher after exercise (main effect of Time, *p* = 0.06).

**TABLE 1:** Heart rate (HR), MAP, and peripheral biomarkers before and after acute aerobic and resistance exercise.

Variable	Aerobic		Resistance			*P*-value	
					Main effect		Interaction
			
	Pre	Post	Pre	Post	Exercise type	Time	
HR, bpm	70 ± 5	116 ± 12***	67 ± 14	104 ± 14*** ^##^	*F*_(1_,_18)_ = 7.20, *p* = 0.015, η_*p*_^2^ = 0.29	*F*_(1_,_18)_ = 355.16, *p* < 0.001, η_*p*_^2^ = 0.95	*F*_(1_,_18)_ = 8.52, *p* = 0.009, η_*p*_^2^ = 0.32
RPE	6 (6–7)	12 (10–13)	6 (6–7)	13 (11–13)	*F*_(1_,_18)_ = 2.77, *p* = 0.114, η_*p*_^2^ = 0.13	*F*_(1_,_18)_ = 345.20, *p* < 0.001, η_*p*_^2^ = 0.95	*F*_(1_,_18)_ = 3.09, *p* = 0.096, η_*p*_^2^ = 0.15
MAP, mmHg	88 ± 4	88 ± 8	88 ± 8	86 ± 8	*F*_(1_,_18)_ = 0.75, *p* = 0.398, η_*p*_^2^ = 0.04	*F*_(1_,_18)_ = 0.67, *p* = 0.423, η_*p*_^2^ = 0.04	*F*_(1_,_18)_ = 1.27, *p* = 0.275, η_*p*_^2^ = 0.07
Adrenaline, pg/mL	37 ± 14	69 ± 23	27 (18–59)	59 ± 28	*F*_(1_,_18)_ = 1.32, *p* = 0.226, η_*p*_^2^ = 0.07	*F*_(1_,_18)_ = 98.89, *p* < 0.001, η_*p*_^2^ = 0.85	*F*_(1_,_18)_ = 1.88, *p* = 0.187, η_*p*_^2^ = 0.10
Noradrenaline, pg/mL	351 ± 90	523 ± 170	377 ± 147	548 (437–674)	*F*_(1_,_18)_ = 2.19, *p* = 0.156, η_*p*_^2^ = 0.11	*F*_(1_,_18)_ = 23.31, *p* < 0.001, η_*p*_^2^ = 0.56	*F*_(1_,_18)_ = 0.65, *p* = 0.430, η_*p*_^2^ = 0.04
Glucose, mg/dL	83 ± 6	80 ± 7	84 ± 8	81 ± 7	*F*_(1_,_18)_ = 0.40, *p* = 0.538, η_*p*_^2^ = 0.02	*F*_(1_,_18)_ = 14.70, *p* = 0.001, η_*p*_^2^ = 0.45	*F*_(1_,_18)_ = 0.05, *p* = 0.822, η_*p*_^2^ = 0.003
Lactate, mmol/L	1.1 ± 0.3	1.2 (1.1–1.4)*	1.1 (1.0–1.2)	2.8 (2.3–3.2)*** ^###^	*F*_(1_,_18)_ = 48.57, *p* < 0.001, η_*p*_^2^ = 0.73	*F*_(1_,_18)_ = 102.62, *p* < 0.001, η_*p*_^2^ = 0.85	*F*_(1_,_18)_ = 36.59, *p* < 0.001, η_*p*_^2^ = 0.67
Cortisol, μg/mL	15 ± 5	11 (10–15)	16 ± 5	12 (9–19)	*F*_(1_,_18)_ = 1.18, *p* = 0.292, η_*p*_^2^ = 0.06	*F*_(1_,_18)_ = 13.31, *p* = 0.002, η_*p*_^2^ = 0.43	*F*_(1_,_18)_ = 0.008, *p* = 0.932, η_*p*_^2^ = 0.000
IGF-1 ng/mL	211 ± 56	209 ± 51	203 ± 45	213 ± 40**	*F*_(1_,_18)_ = 0.10, *p* = 0.752, η_*p*_^2^ = 0.01	*F*_(1_,_18)_ = 3.48, *p* = 0.079, η_*p*_^2^ = 0.16	*F*_(1_,_18)_ = 12.10, *p* = 0.003, η_*p*_^2^ = 0.40
BDNF, pg/mL	27,465 ± 6,281	28,694 ± 7,099	29,094 ± 5,695	29,988 ± 7,488	*F*_(1_,_16)_ = 1.67, *p* = 0.215, η_*p*_^2^ = 0.09	*F*_(1_,_18)_ = 3.98, *p* = 0.063, η_*p*_^2^ = 0.20	*F*_(1_,_18)_ = 0.069, *p* = 0.796, η_*p*_^2^ = 0.004

*Values are mean ± standard deviation or median (interquartile range).*

***p < 0.001, **p < 0.01, *p < 0.05 vs. pre. ^###^p < 0.001, ^##^p < 0.01 vs. aerobic exercise.

*HR, heart rate; RPE, ratings of perceived exertion; MAP, mean arterial pressure; IGF-1, Insulin-like growth hormone factor 1; BDNF, Brain-derived neurotrophic factor.*

**FIGURE 2:**
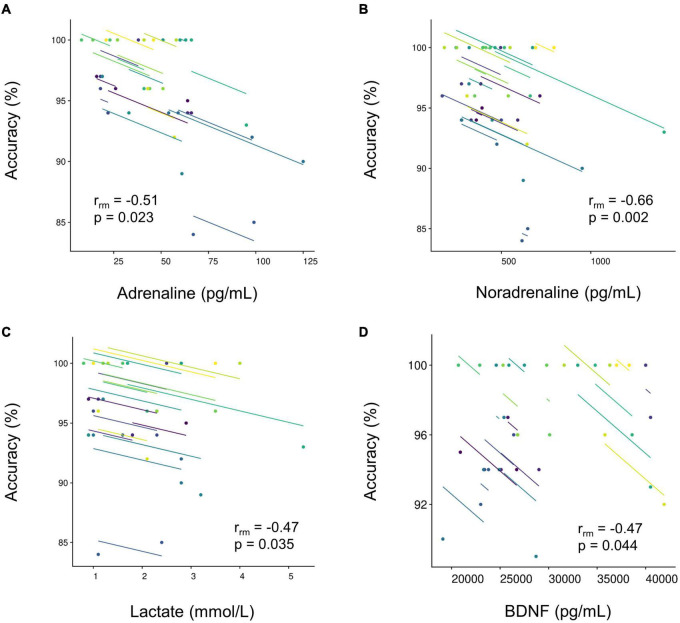
Repeated measure correlations between the accuracy of the Go/No-Go task and peripheral circulating biomarkers [**(A)** adrenaline, *n* = 19; **(B)** noradrenaline, *N* = 19; **(C)** lactate, *N* = 19; and **(D)** brain-derived neurotrophic factor, *N* = 18] in the resistance condition. Plots represent individual data. Lines show the r_*rm*_ fit for the participants. Same colors represent data from the same participants.

Table 2 displays the results of repeated measures correlation analysis. Reduction in RT was not associated with changes in any of the circulating biomarkers (all *p* > 0.05). Conversely, accuracy was associated with changes in adrenaline [r_*rm*_(18) = −0.51, *p* = 0.023], noradrenaline [r_*rm*_(18) = −0.66, *p* = 0.002], lactate [r_*rm*_(18) = −0.47, *p* = 0.035], and BDNF [r_*rm*_(17) = −0.47, *p* = 0.044] in the resistance (Figure 2), but not aerobic (all *p* > 0.05), condition.

**TABLE 2:** Repeated measures correlation between cognitive performance (reaction time and accuracy) and peripheral biomarkers.

Variable	Aerobic		Resistance	
	Reaction time	Accuracy	Reaction time	Accuracy
Adrenaline	r_*rm*_(18) = −0.25, *p* = 0.285	r_*rm*_(18) = 0.24, *p* = 0.31	r_*rm*_(18) = −0.36, *p* = 0.118	r_*rm*_(18) = −0.51, *p* = 0.023*
Noradrenaline	r_*rm*_(18) = −0.30, *p* = 0.199	r_*rm*_(18) = 0.11, *p* = 0.641	r_*rm*_(18) = 0.15, *p* = 0.533	r_*rm*_(18) = −0.66, *p* = 0.002**
Glucose	r_*rm*_(18) = −0.12, *p* = 0.606	r_*rm*_(18) = −0.28, *p* = 0.224	r_*rm*_(18) = −0.10, *p* = 0.67	r_*rm*_(18) = 0.21, *p* = 0.383
Lactate	r_*rm*_(18) = −0.24, *p* = 0.303	r_*rm*_(18) = 0.28, *p* = 0.236	r_*rm*_(18) = −0.18, *p* = 0.437	r_*rm*_(18) = −0.47, *p* = 0.035*
Cortisol	r_*rm*_(18) = 0.12, *p* = 0.605	r_*rm*_(18) = 0.00, *p* = 0.996	r_*rm*_(18) = −0.12, *p* = 0.616	r_*rm*_(18) = 0.28, *p* = 0.229
IGF-1	r_*rm*_(18) = −0.19, *p* = 0.423	r_*rm*_(18) = 0.03, *p* = 0.912	r_*rm*_(18) = −0.07, *p* = 0.762	r_*rm*_(18) = −0.40, *p* = 0.081
BDNF	r_*rm*_(17) = −0.24, *p* = 0.332	r_*rm*_(17) = 0.33, *p* = 0.163	r_*rm*_(17) = −0.01, *p* = 0.956	r_*rm*_(17) =−0.47, *p* = 0.044*

**p < 0.01, *p < 0.05.

## Discussion

The major findings of this study were: (1) executive performance improved (i.e., reduced RT) after acute RPE-matched aerobic and resistance exercise, while accuracy of the executive task tended to be impaired after resistance exercise; (2) acute physical exercise resulted in alterations in peripheral biomarkers; (3) reduced RT was not correlated with alterations in peripheral biomarkers; and (4) accuracy was associated with increases in peripheral adrenaline, noradrenaline, lactate, and BDNF after resistance, but not aerobic, exercise.

Previous studies using electroencephalogram suggest increases in arousal following both aerobic (Kamijo et al., 2004) and resistance (Tsai et al., 2014) exercise. Acute physical exercise has also been implicated in altering brain circuits involving neurotransmitters (Pontifex et al., 2019; McMorris, 2021). This suggests that executive improvement after acute physical exercise may be related to increased central neurochemical activity. We observed reduced RTs and increases in adrenaline and noradrenaline after both aerobic and resistance exercise. As the increases in both adrenaline and noradrenaline were comparable after aerobic and resistance exercise, it is plausible that the activation of the sympathoadrenomedullary system was also relatively similar following the two types of exercise. Nevertheless, alterations in RTs were not correlated with alterations in peripheral adrenaline and noradrenaline. The catecholamine hypothesis describing the exercise-cognition interaction is intriguing (Cooper, 1973). However, the specific mechanism(s) by which acute physical exercise improves executive performance warrant further investigation. We observed a tendency for accuracy to be decreased after resistance exercise (*p* = 0.054). Furthermore, lower accuracy was associated with a greater increase in peripheral adrenaline and noradrenaline after resistance exercise. Although we did not assess adrenaline and noradrenaline level directly in the brain, excess noradrenaline appears to impair prefrontal cortex function including cognitive function in the brain (Arnsten, 2011). Therefore, the present findings may suggest that greater sympathoexcitation has the potential to be associated with accuracy in a Go/No-Go task.

Executive performance improves during exercise after skipping breakfast (Komiyama et al., 2016), which suggests that energy substrates may compensate for reduced availability of blood glucose. It is possible that lactate is used by the brain (Quistorff et al., 2008), and increases in blood lactate concentration appear to provide energy that contributes to improvements in attentional and executive performance after high intensity exercise (Tsukamoto et al., 2016; Hashimoto et al., 2018; Herold et al., 2022). Conversely, Coco et al. (2009, 2020) have shown that increase in blood lactate concentration have adverse effects on attentional and executive performances. We observed no associations between reduction in RT and alterations in lactate or glucose. The findings are inconsistent with others (Tsukamoto et al., 2016; Hashimoto et al., 2018; Herold et al., 2022). It is likely that these heterogeneous findings are attributable to methodological differences. First, the exercise intensity was higher (high intensity interval exercise: 80—90% and 50–60% of maximal workload) in previous studies (Tsukamoto et al., 2016; Hashimoto et al., 2018), compared to the intensity used in the current study (40% V̇O_2*peak*_). Given that blood lactate substantially increases after high intensity, contribution of blood lactate to cognitive improvement after acute physical exercise may be dependent on the degree of increase in blood lactate. Second, Hashimoto et al. (2018) directly measured brain blood lactate uptake, while blood lactate was measured from the antecubital vein in the present study. This may also explain the lack of a significant correlation between executive improvement and increase in blood lactate.

In the resistance condition, the trend toward impaired accuracy was modestly associated with blood lactate. Blood lactate uptake in the brain becomes significantly elevated when arterial lactate increases, for instance, in response to strenuous physical exercises (Quistorff et al., 2008). In the present study, increases in blood lactate was limited up to around 3 mmol L^–1^ in most cases. Hence, the amount of blood lactate uptake in the brain is presumably minimal, and it is less likely that blood lactate directly impaired the accuracy. Our findings suggest that there is a large inter-individual variability in accuracy of a task probing executive functioning after acute resistance exercises (see Figure 1D). Furthermore, accuracy seems to be more impaired in individuals who showed more pronounced physiological alterations (i.e., higher level of peripheral blood lactate) in response to an acute bout of resistance exercises (see also Figure 2C). In general, the latter finding fits to the observations of Coca and colleagues who reported that high levels of lactate have detrimental effects on attentional and executive performance (Coco et al., 2009, 2020). However, given the inconclusive results in the literature, further studies are warranted to investigate whether lactate acts as a possible mediator of exercise-induced changes in executive performance.

The HPA system is sensitive to acute physiological stress including exercise (Gaffey et al., 2016; Williams et al., 2019). In the present study, cortisol decreased after both aerobic and resistance exercise. Hill et al. (2008) reported that low intensity aerobic exercise (i.e., 40% maximal oxygen uptake) decreased circulating cortisol level (Hill et al., 2008). Tsai et al. (2014) also reported decrease in cortisol level after moderate resistance exercise (Tsai et al., 2014). The present results are in line with these observations. We observed no association between executive improvement and in the change in cortisol after acute aerobic or resistance exercise. Tsai et al. (2014) suggested that arousal level might be modulated by alteration in cortisol. However, alterations in cortisol were not associated with alterations in executive performance. These findings suggest cortisol may not be directly associated with executive performance after acute physical exercise.

Consistent with the literature acute resistance, but not aerobic, exercise increased peripheral IGF-1 (Rubin et al., 2005; Rojas Vega et al., 2010; Tsai et al., 2014; Tsai et al., 2018). BDNF also tended to increase after both aerobic and resistance exercise. Although this is not always the case, previous studies have reported that BDNF increases after exercise at higher intensities (Ferris et al., 2007; Winter et al., 2007). Therefore, given the relatively low exercise intensity of the present study, the absence of significant increases in BDNF are perhaps not surprising. We observed no association between executive performance and alterations in IGF-1 and BDNF. Previous studies reported no association between alterations in IGF-1 and executive performance after acute resistance exercise (Tsai et al., 2014, 2018). Similarly, increases in IGF-1 after exhaustive exercise have previously been shown to be unrelated with executive performance (Sudo et al., 2017) and alterations in BDNF were not associated with executive performance and neurophysiological variables after acute physical exercise (Tsai et al., 2016). In contrast, significant associations were observed between alteration in BDNF and performance in executive and memory tasks after acute physical exercise (Winter et al., 2007; Lee et al., 2014; Skriver et al., 2014). Thus, although alterations in BDNF appears not to be directly associated with improvement in executive performance after acute physical exercise, BDNF may contribute to memory performance. Given that IGF-1 and BDNF are associated with angiogenesis, synaptogenesis, and neurogenesis after long-term exercise (Cotman and Berchtold, 2002; Voss et al., 2011; Nieto-Estevez et al., 2016), it is reasonable to think that alterations in IGF-1 or BDNF may play a minor role in executive performance following acute physical exercise. Nevertheless, further studies are required to clarify the contribution of IGF-1 and BDNF to cognitive performance (e.g., memory performance) after acute physical exercise. In the resistance condition, the trend toward an impairment in accuracy was moderately associated with BDNF. Previous studies indicated that increase in BDNF is dependent on exercise intensity (Ferris et al., 2007; Winter et al., 2007). Thus, like the association between impaired accuracy and blood lactate, accuracy could be more impaired in those individuals showing more pronounced alterations in specific biomarkers in response to a given exercise intensity.

The present study was not without limitation. First, despite attempting to match exercise intensity between the aerobic and resistance exercise, there were significant differences in HR (∼12 beats min^–1^). We also found inter-individual differences in HR across both conditions. Second, we observed a trend toward an impairment in accuracy and the association between accuracy and peripheral biomarkers only in the resistance condition. Given that dose-response is important to examine exercise-cognition interaction (Herold et al., 2019, 2020), the results may be attributable to these differences in relative exercise intensity. Third, since we did not include a control condition, we cannot rule out the possibility that time-related factors including practice effects and circadian change of peripheral biomarkers. However, the participants were familiarized with the cognitive task and exercise duration was only 30 min. Thus, the effects may be small, if any. Fourth, we estimated sample size based on the primary outcome (i.e., RT). This might lead to low statistical power to detect the association between cognitive performance and peripheral biomarkers. Indeed, we observed no significant correlations between the reductions in RTs and peripheral biomarkers following acute aerobic and resistance exercise. The present results suggest that these peripheral circulating biomarkers are not capable of indicating the level of executive performance after exercise. However, the effects of acute physical exercise on cognitive performance are multifaceted and probably determined by the integration of several physiological factors. Perhaps, some of these peripheral biomarkers may contribute to executive improvement in a synergistic manner. Fifth, we did not take consider the impact of other potential confounding factors including nutritional status, habitual physical activity, and psychological factors (e.g. motivation, concentration, and fatigue). Finally, we collected venous and capillary blood samples immediately after aerobic and resistance exercise. The timing of the measurement is commonly used in the literature. However, concentrations of peripheral biomarkers appear to change depending on the time elapsed after physical exercises (e.g., Griffin et al., 2011; Gregory et al., 2013; Hashimoto et al., 2018), and the association between executive performance and peripheral biomarkers are likely to be influenced by the timing of measurement (e.g., immediately or 15 min after exercise). This should be considered in future studies.

## Conclusion

In conclusion, despite observing a reduction in RT in the Go/No-Go task following both aerobic exercise and resistance exercise, we observed no significant correlations between these reductions and peripheral adrenaline, noradrenaline, glucose, lactate, cortisol, IGF-1, or BDNF. These results suggest that alterations in these peripheral circulating biomarkers do not directly contribute to improved RT after aerobic and resistance exercise. However, greater sympathoexcitation, reflected by greater increase in noradrenaline, may be associated with a tendency for a reduction accuracy after acute resistance exercise.

## Data Availability Statement

The raw data supporting the conclusions of this article will be made available by the authors, without undue reservation.

## Ethics Statement

The studies involving human participants were reviewed and approved by the Ethics Committee of Fukuoka University. The patients/participants provided their written informed consent to participate in this study.

## Author Contributions

SA and TK contributed to the conception and design of the study and drafted the manuscript. SA, TK, YT, and MS acquired the data and performed the data analysis. JC, YU, and YH edited and revised the manuscript. All authors interpreted the results, read, and approved the final version of the manuscript.

## Conflict of Interest

The authors declare that the research was conducted in the absence of any commercial or financial relationships that could be construed as a potential conflict of interest.

## Publisher’s Note

All claims expressed in this article are solely those of the authors and do not necessarily represent those of their affiliated organizations, or those of the publisher, the editors and the reviewers. Any product that may be evaluated in this article, or claim that may be made by its manufacturer, is not guaranteed or endorsed by the publisher.
