# Supplementary material for: Cognitive Improvement After Aerobic and Resistance Exercise Is Not Associated With Peripheral Biomarkers
Source: Front Behav Neurosci. 2022 Mar 15;16:853150. doi: 10.3389/fnbeh.2022.853150 (PMC8967356; doi:10.3389/fnbeh.2022.853150)
Supplement: Supplementary file 1 [file Table_1.docx]

Supplemental Table 1 Resistance exercise program using the elastic band.

| Target muscle | Exercise program | | | | | |
| --- | --- | --- | --- | --- | --- | --- |
| Shoulder | Shoulder stretch | Front raise | Pull back | Two arms upright row | External rotation | Internal rotation |
| Chest | Press | High press | Low press | Fly | Push up | Chest stretch |
| Back | Two arms external rotation | Bent over row | One arm upright row | Back stretch | Lat pull down | Seated row |
| Arms | Curl | Triceps | One arm triceps | Wrist curl | Reverse wrist curl | Pronation |
| Abdomen | Knee up | Standing crunch | Side bend | Truck twist | Crunch | Back across pull |
| Hip | Deadlift | Abduction | Hip extension | Wide squat | Hamstring stretch | Hip stretch |
| Legs | Leg extension | Leg curl | Squat | Lunge | Calf raise | Leg stretch |
